# Association Between Pain and Fall Worry Among Community-Dwelling Older People With Cognitive Impairment in the United States

**DOI:** 10.1093/geroni/igad100

**Published:** 2023-09-16

**Authors:** Yuanjin Zhou, Namkee G Choi, Tatiana Sadak, Nayanika Ghosh, Elizabeth A Phelan

**Affiliations:** Steve Hicks School of Social Work, University of Texas at Austin, Austin, Texas, USA; Steve Hicks School of Social Work, University of Texas at Austin, Austin, Texas, USA; School of Nursing, University of Washington, Seattle, Washington, USA; Department of Speech, Language, and Hearing Sciences, University of Texas at Austin, Austin, Texas, USA; Division of Gerontology and Geriatric Medicine, School of Medicine, University of Washington, Seattle, Washington, USA; Department of Health Systems and Population Health, School of Public Health, University of Washington, Seattle, Washington, USA

**Keywords:** Community dwelling, Cognitive impairment, Dementia, Fear of falling, NHATS

## Abstract

**Background and Objectives:**

Previous studies have found that pain is associated with fall worry among community-dwelling older people. However, both pain and fall worry are poorly understood and underaddressed among community-dwelling older people with cognitive impairment (CI). It is essential to examine the association between pain and fall worry, and how sociodemographic and health characteristics may shape fall worry among this subgroup.

**Research Design and Methods:**

We used data from the 2015 National Health and Aging Trends Study (analytic sample: *n* = 1150 community-dwelling older people with CI; were self-interviewed; mean age: 81; age range: 65–107). The number of pain sites in the prior month was assessed by presenting a card listing common pain sites (eg, back, knees). Two questions assessed past-month fall worry, “did you worry about falling down” and “did this worry ever limit your activities.” Following descriptive statistics, we fit multinomial logistic regression models to examine the associations between different pain characteristics (number of sites, severity, location) and non-activity-limiting and activity-limiting fall worry.

**Results:**

Non-activity-limiting fall worry was endorsed by 21.1% and activity-limiting fall worry by 13.6% of community-dwelling older people with CI. After adjusting for sociodemographic characteristics and fall-worry-related covariates, multinomial logistic regression analysis found that a greater number of pain sites (relative risk ratio [RRR] = 1.22, 95% Confidence Interval [95% CI] = 1.12–1.33, *p* <.001) and severe pain (RRR = 2.05, 95% CI = 1.12–3.75, *p* = .020) was associated with activity-limiting fall worry. Both lower body (knee, foot, and leg) and upper body (hand, wrist, shoulder, neck, and stomach) pain were found to be associated with a high risk of activity-limiting fall worry.

**Discussion and Implications:**

These findings suggest pain and fall worry are common among community-dwelling older people with CI and can be elicited directly from those who are communicative. Fall prevention for this population should prioritize pain management to mitigate activity-limiting fall worry because activity limitation increases the risk of falls.

**Translational Significance:** Pain and fall worry are potentially modifiable fall risk factors for community-dwelling older people with cognitive impairment (CI). However, both pain and fall worry are poorly understood and underaddressed in this population. Our study highlights the negative impact of pain, especially multisite pain, severe pain, and pain in the lower and upper body, on activity-limiting fall worry. Clinicians working with older people with CI who present with pain, especially those with multisite or severe pain, should consider assessing fall worry and related activity restriction, and vice versa. Pain management should be incorporated into fall prevention interventions for this population.

Older people with cognitive impairment (CI), meaning those with either mild cognitive impairment (MCI) or dementia, have 2–10 times higher risk of falls compared to their age-matched peers without CI (1,2). They also have an approximately threefold increased risk of sustaining a fracture after falls (3), often resulting in hospitalization, nursing home placement, and mortality (4). Moreover, their falls are more likely to incur high costs when hospitalized (5), as they are more likely to experience delirium (6) and in-hospital falls (7) than those without CI. Therefore, fall prevention for community-dwelling older people with CI is a high-priority public health issue (8). However, evidence is limited for effective fall prevention in this population (9). It is critical to identify modifiable factors relevant to this high-risk population to inform the development of effective preventive strategies.

Fall worry (often referred to as fear of falling, a fall-related psychological concern (10)) is a significant fall risk factor among older people. Many age-related conditions, such as chronic illnesses and sensory impairment, may increase older people’s risk of falling (8). Many older people worry about falling, whether or not they have fallen in the past (11). A certain degree of concern may be protective, helping to avoid a fall through avoidance of perceived hazards and undertaking activities at a slower pace (12,13). However, when fall worry limits social and physical activities, it negatively affects older adults’ functional capacity and social relationships and ultimately increases their risk of falling (12,14). Studies that used standardized measures to capture fall worry have found that fear of falling in older people with CI is frequently preserved, especially in MCI and early- and moderate-stage dementia, despite impaired cognitive functioning (15–18). Fall worry leads them to restrict their daily activities (19), including leaving their home, even though they may still have preserved mobility ability (20). These activity reductions can have a series of adverse consequences, including lower quality of life (19), restricted life-space mobility (21), and reduced social engagement (18), all of which further contribute to an increased risk of falls (8,22).

Other studies have found that those with CI, especially those who are frail (23), and have global cognitive impairment (Mini-Mental State Examination score of 23 or lower) (17), sometimes lack sufficient awareness of fall risk, which may result in their engagement in risky behaviors (24). These behaviors may not only result in a higher risk of falls (25) but also increase the care burden for care partners (26). Given the unique challenges of balancing awareness of fall risk and mitigating the negative impacts of fall worry on activity restriction among those with declining cognitive function, it is imperative to understand what factors contribute to their activity-limiting fall worry.

The prevalence of fall worry among community-dwelling older people with CI ranges from 30% to 90%, depending on sample characteristics (especially, the severity of CI, stages, and types of dementia) and assessment approaches (16,17,27,28). Although there is a large body of research on the correlates of fall worry among older people without CI (10), previous studies suggest that older people with and without CI may have different experiences of fall worry (16,28). As there is limited research examining the etiology and correlates of fall worry among this population, further investigation is warranted.

Some studies have found that people living with MCI and dementia with Lewy bodies are more likely to experience a higher degree of fall worry than people living with Alzheimer’s disease (16,27). A few other studies reported that older age, female gender, hearing and visual impairment, lower educational attainment, living alone, depression, history of falls, and use of mobility devices were associated with general fall worry for community-dwelling older people with varying degrees of cognitive functions (18,21,28–31). However, these findings were based on only a limited number of studies using comparatively small samples and including limited covariates. Few studies have examined factors associated with activity-limiting fall worry among older people with CI.

One potential modifiable risk factor of fall worry for older people with CI is pain. Different pain characteristics (ie, number of sites, severity, location) are associated with fall worry among the general older people population (10). It is estimated that approximately half of community-dwelling older people with CI experience pain (32), which is often under-recognized and undertreated (33). Pain contributes to numerous adverse outcomes, such as behavioral and psychological symptoms (34), depression (35), lower quality of life (36), use of psychotropic medications (37), decreased activity engagement (38), and high levels of disability and mortality (39). However, to our knowledge, no study has examined whether and how pain is associated with fall worry among those with CI. Such understanding can help develop more effective integrative interventions to address the unmet needs of older people with CI in managing pain and fall worry.

For the present study, based on a nationally representative sample of community-dwelling U.S. Medicare beneficiaries aged 65+ years with possible or probable dementia, as determined by the validated algorithm in the National Health and Aging Trend Study (NHATS) technical papers (40), we examined (i) the prevalence of activity-limiting and nonactivity-limiting fall worry among older people with CI, and (ii) the relationships between different characteristics of pain (number of sites, severity, location) and fall worry (no fall worry, nonactivity-limiting fall worry, and activity-limiting fall worry). The study hypotheses were that (H1) a greater number of pain sites and (H2) severe pain in any location would be associated with activity-limiting fall worry after controlling for covariates. We also hypothesized that (H3) pain in the lower body (knee, foot, leg), hip, and back would be associated with activity-limiting fall worry. Covariates, based on previous research findings (18,21,28–31), include cognitive status, past-year fall incidents, number of chronic illnesses, past-month mobility device use, hearing and visual impairment, and sociodemographic characteristics.

The study findings will shed light on the associations between pain and fall worry among community-dwelling older people with CI and how sociodemographic backgrounds, various disabilities, and health conditions may shape their fall worry. This information will inform the development and implementation of fall risk prevention programs tailored to this population with different characteristics.

## Method

### Data

Analyses herein were conducted using the public use data file of the 2015 (Round 5) NHATS. The NHATS is a longitudinal, nationally representative, prospective cohort study sponsored by the National Institute on Aging through a cooperative agreement with the Johns Hopkins Bloomberg School of Public Health. The NHATS was first conducted in 2011 (Round 1) with a sample representative of U.S. Medicare beneficiaries aged 65+ as of September 30, 2010, who resided in the community in a private residence (their own or someone else’s) or residential care (but not nursing homes) communities, with an oversampling of non-Hispanic Blacks and those aged 85 years and older using a stratified, 3-stage sampling design (41). These participants undergo annual in-person interviews that collect detailed information on the disablement process and its consequences. Replenishment occurred in Round 5, with a sample drawn from the Medicare enrollment database serving as the sampling frame as of September 30, 2014 (42). Although the NHATS now has 12 Rounds (2011–2022), Round 5 data with replenishment allowed the study to maximize the sample size of community-dwelling older people with CI and provided the most recent estimation on the population level.

### Analytic Sample

The analytic sample included 1150 participants, representing 4.6 million community-dwelling Medicare beneficiaries living with CI who were communicative. Participants were characterized as having possible or probable dementia through NHATS’s dementia classification (40), lived in community settings, were self-interviewed, and provided fall worry data in 2015. Supplementary Figure 1 shows the number of observations excluded at each stage. As our interest was in the experience of fall worry and pain described by older people with CI, we excluded 322 participants who relied on a proxy respondent to respond to survey questions relevant to the current study. Participants used proxy respondents for the following reasons: Participants’ dementia/cognitive/mental impairment reported by proxy (*n* = 214), illness/major physical health issues (*n* = 124), speech impairment (*n* = 30), hearing impairment (*n* = 60), language barriers (*n* = 32), temporary unavailability (*n* = 5), or other reasons (*n* = 19). Our final sample included 706 with possible dementia and 444 with probable dementia, as defined later.

The classification of possible dementia was defined by a score of 1.5 standard deviations below the mean in only 1 of 3 domains of the cognitive functioning test, including memory (self-rated whether memory interferes with daily activities and immediate and delayed 10-word recall), orientation (date, month, year, day of the week, naming president and vice president), and executive function (clock drawing test) (40). Probable dementia was classified in 2 ways: (i) a self-report of medical provider-diagnosed dementia or Alzheimer’s disease; or (ii) a cognitive test score 1.5 standard deviations below the mean in 2 or more domains out of 3 total (memory, orientation, and executive function). This algorithm was reported to have an overall sensitivity of 71.8% and specificity of 83.7% for CI and dementia in the Aging, Demographics, and Memory Study, a subsample of another nationally representative cohort employing gold-standard diagnostic assessment (40). Herein, we refer to those with either possible or probable dementia as “older people with cognitive impairment.”

### Dependent Variable: Fall Worry

Fall worry was measured by combining 2 items: “In the last month, did you worry about falling down?” (yes/no); and “(for those who responded ‘yes’) In the last month, did this worry ever limit your activities?” (yes/no). “Falling down” was defined as any fall, slip, or trip, which resulted in a loss of balance and landing on the floor or ground or at a lower level. Responses to these 2 items were used to generate a 3-level ordinal variable, “no fall worry,” “nonactivity-limiting fall worry,” and “activity-limiting fall worry.” This ordinal variable of fall worry has been used as a dependent variable in previous studies of older people with and without CI (11,43).

### Independent Variable: Pain Characteristics

We included multiple independent variables representing different characteristics of pain, including the number of pain sites, severity, and location.

(1) The number of pain sites: Participants were asked to identify the location of pain in the last month from a card listing back, hips, knees, legs, feet, hands, wrists, arms, shoulders, stomach, head, neck, and any other location(s). Although persons with CI may have challenges verbalizing pain location (44), those with mild-to-moderate dementia can self-report pain location(s) using their own body, a doll, a diagram (45), or a body map from the McGill Pain Questionnaire (46), which was similar to the pain location card used in the NHATS. The side of the body where the pain occurred was not recorded. For descriptive purposes, the number of pain sites (0–13) was also grouped as no pain site, single pain site, and multiple pain sites (≥2).
(2) Pain severity: For participants who reported at least 1 pain site, we examined 2 questions regarding pain severity: “In the last month, have you been bothered by pain (yes/no)”; and “(for those who answered ‘yes’) Did bothersome pain limit your activities in the last month” (yes/no). Responses to these 2 items were used to create a 3-level ordinal variable of pain severity: Mild pain (have at least 1 pain site, but the pain is not bothersome), moderate pain (bothersome but not activity-limiting), and severe pain (activity-limiting pain). Several studies have demonstrated that persons with mild-to-moderate dementia can self-report pain using a similar verbal descriptor scale with good reliability and validity (33,47).
(3) Pain location: For participants who reported at least 1 pain site, we examined their pain at a specific location using 12 Pain location variables: pain in the back, hip, knee, leg, foot, hand, wrist, arm, shoulder, stomach, head, and neck.

### Covariates

We included sociodemographic and health status variables that might be associated with fall worry for community-dwelling older people with CI based on previous literature (18,21,28–31). Sociodemographic variables were (i) age group (aged 65–74, 75–84, 85+ years); (ii) gender (female vs male); (iii) race/ethnicity (non-Hispanic White, non-Hispanic Black, Hispanic, all other); (iv) education level (<12, 12, >12 years); (v) marital status (married/partnered vs not married); and (vi) living arrangement (living alone vs living with others).

Health status variables included (i) cognitive status: Possible dementia and probable dementia based on the validated NHATS algorithm (40,48); (ii) past-year fall incidents: Measured by t2 questions “In the past 12 months, have you fallen down” (yes or no); those who answered yes were asked, “In the last 12 months, have you fallen down more than one time”; based on the responses to these 2 questions, fall status was coded as “no fall,” “single fall,” and “2 or more falls”; (iii) number of chronic illnesses diagnosed by a health care professional, ranging from 0 to 9 (heart attack, heart disease, hypertension, arthritis, osteoporosis, diabetes, lung disease, stroke, and hip fracture); (iv) past-month mobility device use: participants were asked “In the last month, did you use a cane, walker, wheelchair, or scooter to help you get around more easily, safely, or on your own?” (yes or no); (v) hearing and visual impairment: Participants were considered to have visual impairment if they reported blindness, or an inability to see well enough to recognize someone across the street, to watch television across the room, or to read newspaper print (while wearing glasses/contacts). Participants were considered to have a functional hearing impairment if they reported deafness, hearing aid use, an inability to hear well enough to use the telephone, or an inability to carry on a conversation in a room with the television or radio playing. Depression/anxiety symptoms (over the last month; assessed with the Patient Health Questionnaire-4 [PHQ-4]) (49), self-reported balance, and the number of limitations in activities and instrumental activities of daily living (ADLs/IADLs), ranging from 0 to 12 (feeding, bathing, toileting, dressing, bed transfer, moving inside the house, go outside, doing laundry, shopping, preparing meals, taking medication, and managing money) were examined for descriptive purpose only.

### Statistical Analysis

All analyses were conducted with Stata/MP 17’s svy function to account for NHATS’ stratified, multistage sampling design following the NHATS technical paper #16 (50), and subpop command to conduct analysis for the targeted population. First, Chi-square and ANOVA tests were conducted to compare 3 fall worry groups (no worry, nonactivity-limiting worry, and activity-limiting worry) on sociodemographic, health status, and pain characteristics. Second, for those with at least 1 pain site, we compared pain severity and pain location by type of fall worry. Third, we fit a series of multinomial logistic regression models to test our hypotheses regarding the association of the number of pain sites, pain severity, and pain location with fall worry. We performed a series of post hoc analyses to assess the impact of cognitive status. These analyses divided participants into 2 dementia subgroups: Possible dementia (*n* = 706) and probable dementia (*n* = 444). All models included covariates described in the measures section. We used the variance inflation factor (VIF) with a cutoff point of 2.50 (51) from a linear regression model to assess multicollinearity among covariates. VIF scores suggested that multicollinearity was not a concern. Multinomial logistic regression results are presented as adjusted relative risk ratios (RRR) with 95% confidence intervals (95% CI). Significance was set at *p* < .05.

## Results

### Fall Worry, Cognitive Status, and Pain Experiences

Among community-dwelling older people with CI: 65.3% reported no fall worry, 21.1% reported nonactivity-limiting fall worry, and 13.6% reported activity-limiting fall worry (Table 1). Of those who reported nonactivity-limiting fall worry, 69.2% had possible dementia, and 30.8% had probable dementia. Of those who reported activity-limiting fall worry, 57.3% had possible dementia, and 42.7% had probable dementia. The mean number of reported pain sites was 2.5 (standard error [*SE*] = 0.1) for all older people with CI; over half (54.2%) experienced pain in multiple sites. Among those who experienced nonactivity-limiting fall worry, the mean number of reported pain sites was 3.0 (*SE* = 0.2); about 80% reported at least 1 pain site and 65.1% had multiple pain sites. Among those who experienced activity-limiting fall worry, the mean number of reported pain sites was 4.2 (*SE* = 0.3); 90% had at least 1 pain site, and nearly 80% had multisite pain.

**Table 1.** Sociodemographic Characteristics and Health Status by Fall Worry Among Community-Dwelling Older People With Cognitive Impairment (*N* = 1 150)^*^

Variable	All, *N* = 1 150 (100%)	(1) No Fall Worry, *n* = 754 (65.3%)	(2) Nonactivity-Limiting Fall Worry, *n* = 235 (21.1%)	(3) Activity-Limiting Fall Worry, *n* = 161 (13.6%)	*p* Value^†^ (among all 3 groups)	*p* Value (Group 1 and Group 2)	*p* Value (Group 2 and Group 3)
Cognitive status (%)							
Possible dementia	66.1	67.0	69.2	57.3	.103	.614	**.036**
Probable dementia	33.9	33.0	30.8	42.7			
The number of pain sites (mean, *SE*^‡^)	2.5 (0.1)	2.0 (0.1)	3.0 (0.2)	4.2 (0.3)	**<.001**	**<.001**	
Pain site group (%)							
No pain site	26.4	31.4	20.1	12.2	**<.001**	**<.001**	.087
Single pain site	19.4	22.7	14.8	10.6			
Multiple pain sites (2–13)	54.2	45.9	65.1	77.2			
Age group (years, %)							
65–74	33.9	37.2	31.5	21.8	**.012**	.131	.078
75–84	41.2	40.1	38.4	50.4			
85+	25.0	22.8	30.1	27.8			
Gender (%)							
Male	50.5	55.9	40.9	39.4	**.002**	**.003**	.839
Female	49.5	44.1	59.1	60.6			
Race/ethnicity (%)							
Non-Hispanic White	65.2	63.0	69.7	68.9	**.028**	**.046**	.941
Non-Hispanic Black	15.1	17.9	10.6	9.2			
Hispanic	14.1	12.3	16.7	18.4			
Other^§^	5.6	6.9	3.0	3.5			
Education (%)							
<12 years	37.0	33.9	47.0	36.4	**.030**	**.006**	.184
12 years	31.9	34.3	25.9	30.3			
>12 years	31.0	31.9	27.1	33.3			
Marital status (%)							
Married/partnered	45.5	48.6	41.6	36.7	**.030**	.110	.379
Not married	54.5	51.4	58.4	63.3			
Live alone (%)							
Yes	31.0	29.9	34.0	31.5	.485	.260	.553
No	69.0	70.1	66.0	68.5			
Past-month PHQ-4 score (mean, *SE*)	2.4 (0.1)	1.8 (0.1)	3.3 (0.2)	4.4 (0.3)	**<.001**	**<.001**	**.003**
No. of medical conditions (%)							
0–1	24.1	27.9	17.5	16.1	**<.001**	**<.001**	.860
2–3	45.7	48.4	41.5	39.4			
4–9	30.3	23.7	41.0	44.5			
History of falls in 2015 (%)							
No fall	62.5	70.9	50.7	40.2	**<.001**	**<.001**	.082
Single fall	19.9	18.2	24.3	21.3			
Two or more falls	17.7	10.9	25.0	38.5			
Past-month mobility device use (%)							
Yes	40.4	30.6	48.2	75.0	**<.001**	**<.001**	**<.001**
No	59.6	69.5	51.8	25.0			
Self-report balance impairment (%)							
Yes	42.6	28.5	60.6	82.1	**<.001**	**<.001**	**.001**
No	57.4	71.5	39.4	18.0			
Vision impairment (%)							
Yes	11.1	7.5	18.2	17.4	**<.001**	**<.001**	.842
No	88.9	92.5	81.8	82.6			
Hearing impairment (%)							
Yes	16.2	14.6	21.1	16.5	.089	**.020**	0.321
No	83.8	85.4	78.9	83.5			
Activity limitations (mean, *SE*)	1.9 (0.1)	1.4 (0.1)	2.2 (0.2)	4.2 (0.2)	**<.001**	**<.001**	**<.001**

*Notes*: NHATS = National Health and Aging Trend Study; PHQ-4 = Patient Health Questionnaire-4.

^*^Percentages were weighted to account for the NHATS survey design and to produce nationally representative estimates.

^†^ *p* Values were calculated with Pearson χ^2^ tests for categorical variables and ANOVA for the continuous variables. *p* Values of <.05 were bolded.

^‡^ *SE* = Standard error.

^§^“Other” category includes American Indian, Asian, Native Hawaiian, and multiracial.

Table 2 shows that, among those with at least 1 pain site, 24.3% experienced mild pain, 31.9% experienced moderate pain, and 43.8% experienced severe pain. Of those who experienced at least 1 pain site and nonactivity-limiting fall worry, 45.8% had severe pain; for those who experienced activity-limiting fall worry, 67.8% had severe pain. The most commonly reported pain locations were in the back (50.8%), knee (39.9%), leg (38.2%), shoulder (33.7%), and foot (29.5%).

**Table 2.** Pain Severity and Pain Sites by Fall Worry Among Community-Dwelling Older People With Cognitive Impairment and At Least 1 Pain Site (*N* = 835)

Variable	All, *N*=835 (100%)	(1) No Fall Worry, *n* = 512 (60.7%)	(2) Nonactivity-Limiting Fall Worry, *n* = 185 (23.0%)	(3) Activity-Limiting Fall Worry, *n* = 138 (16.3%)	*p* Value^†^ (among all 3 groups)	*p* Value (Group 1 and Group 2)	*p* Value (Group 2 and Group 3)
Pain severity (%)							
Mild	24.3	27.6	21.1	16.4	**<.001**	.195	**.010**
Moderate	31.9	35.8	33.1	15.8			
Severe	43.8	36.5	45.8	67.8			
Pain sites (%)							
1. Back pain	50.8	47.4	51.1	63.2	.067	.586	.085
2. Hip pain	25.8	21.8	29.0	36.3	**.010**	.111	.232
3. Knee pain	39.9	33.5	43.0	59.2	**<.001**	.056	**.009**
4. Foot pain	29.5	24.9	31.2	43.8	**.006**	.208	.078
5. Hand pain	26.2	22.1	27.3	39.9	**<.001**	.185	**.005**
6. Wrist pain	12.1	8.2	14.9	22.3	**.002**	.059	.107
7. Shoulder pain	33.7	28.2	41.6	43.1	**.006**	**.010**	.833
8. Head pain	19.9	16.1	24.7	27.6	**.021**	**.029**	.662
9. Neck pain	23.2	19.0	26.3	34.1	**.013**	.104	.190
10. Arm pain	17.0	12.9	21.0	26.4	**.005**	**.019**	.376
11. Leg pain	38.2	32.3	39.4	57.3	**<.001**	.161	**.010**
12. Stomach pain	15.7	12.8	18.1	23.0	.051	.186	.341

*Note*: *p* Values were calculated with Pearson χ^2^ tests.

### Comparisons by the Level of Fall Worry

Table 1 illustrates how sociodemographic characteristics, health status, and the number of pain sites vary by fall worry. A higher number of pain sites (no fall worry vs nonactivity-limiting fall worry: *p* < .001; nonactivity-limiting fall worry vs activity-limiting fall worry: *p* = .002), a higher PHQ-4 score (*p* < .001; *p* = .003), past-month mobility device use (*p* < .001; *p* < .001), self-reported balance impairment (*p* < .001; *p* = .001), and more activity limitations (*p* < .001; *p* < .001) were significantly associated with both types of fall worry. Analyses showed that compared to those who had no fall worry, those who had nonactivity-limiting fall worry included significantly larger proportions of individuals who were female (nonactivity-limiting fall worry: 59.1% vs no fall worry: 44.1%, *p* = .003), non-Hispanic White (69.7% vs 63.0%, *p* = .046), and having multiple pain sites (65.1% vs 45.9%, *p* < .001), <12 years of education (47.0% vs 33.9%, *p* = .006), 4+ medical conditions (41.0% vs 23.7%, *p* < .001), a fall history (single fall and multiple falls; 49.3% vs 29.1%, *p* < .001), visual impairment (18.2% vs 7.5%, *p* < .001), and hearing impairment (21.1% vs 14.6%, *p* = .020). A larger proportion of those with activity-limiting fall worry had probable dementia compared to those with nonactivity-limiting fall worry (activity-limiting fall worry: 42.7% vs nonactivity-limiting fall worry: 30.8%, *p* = .036).

Table 2 shows how pain severity and pain location vary by fall worry among those who have at least 1 pain site. Compared to those with no fall worry, those who had nonactivity-limiting fall worry included significantly higher proportions of people who experienced pain in the shoulder (nonactivity-limiting fall worry: 41.6% vs no fall worry: 28.2%, *p* = .010), head (24.7% vs 16.1%, *p* = .029), and arm (21.0% vs 12.9%, *p* = .019). Compared to those who experienced nonactivity-limiting fall worry, those who had activity-limiting fall worry included significantly higher proportion of people having severe pain last month (activity-limiting fall worry: 67.8% vs nonactivity-limiting fall worry: 45.8%, *p* = .010) and people who reported pain in the knee (59.2% vs 43.0%, *p* = .009), hand (39.9% vs 27.3%, *p* = .005), and leg (57.3% vs 39.4%, *p* = .010).

### Associations of Fall Worry With the Number of Pain Sites

Table 3 shows that the number of pain sites is significantly associated with the risk of activity-limiting fall worry. With 1 additional pain site, the relative risk of experiencing activity-limiting fall worry compared to no fall worry would increase by a factor of 1.22 (RRR = 1.22, 95% CI = 1.12–1.33, *p* < .001), and the relative risk for experiencing activity-limiting fall worry compared to nonactivity-limiting fall worry would increase by a factor of 1.14 (RRR = 1.14, 95% CI = 1.04–1.24, *p* = .006). The results support H1.

**Table 3.** Associations of Fall Worry With Number of Pain Sites: Multinomial Logistic Regression Results

Variable	(2) Nonactivity-Limiting Fall Worry vs (1) No Fall Worry	(3) Activity-Limiting Fall Worry vs (1) No Fall Worry	(3) Activity-Limiting Fall Worry vs (2) Nonactivity-Limiting Fall Worry
	Adjusted RRR (95% CI)	Adjusted RRR (95% CI)	Adjusted RRR (95% CI)
Number of pain sites	1.08 (0.99–1.17)	1.22 (1.12–1.33)***	1.14 (1.04–1.24)**
Probable dementia (vs mild cognitive impairment/dementia)	0.73 (0.47–1.15)	1.09 (0.66–1.79)	1.48 (0.88–2.51)
Age group: vs 65–75			
75–85	0.94 (0.57–1.55)	1.87 (1.03–3.39)*	2.00 (1.06–3.77)*
85+	1.04 (0.64–1.70)	1.48 (0.81–2.69)	1.42 (0.78–2.58)
Female (vs male)	1.70 (1.09–2.65)*	1.39 (0.77–2.51)	0.82 (0.41–1.64)
Race/ethnicity (vs non-Hispanic White)			
Non-Hispanic Black	0.45 (0.25–0.83)*	0.43 (0.25–0.73)**	0.95 (0.42–2.12)
Hispanic	1.00 (0.52–1.90)	1.71 (0.83–3.55)	1.72 (0.72–4.13)
Other	0.14 (0.03–0.61)**	0.52 (0.16–1.74)	3.78 (0.49–29.06)
Education (vs <12 years)			
12 years	0.43 (0.24–0.75)**	0.99 (0.53–1.85)	2.31 (1.16–4.60)*
>12 years	0.54 (0.32–0.90)*	1.66 (0.84–3.29)	3.09 (1.34–7.17)**
Married/partnered (vs not married)	1.05 (0.57–1.92)	0.64 (0.31–1.30)	0.61 (0.28–1.31)
Live alone (vs living with others)	1.45 (0.82–2.57)	0.98 (0.52–1.85)	0.68 (0.39–1.17)
No. of medical conditions (vs 0–1)			
2–3	1.09 (0.67–1.79)	0.88 (0.40–1.96)	0.81 (0.33–1.97)
4–9	1.76 (0.94–3.30)	1.14 (0.51–2.55)	0.65 (0.26–1.65)
Past-year fall incident (vs 0 fall)			
Single fall	1.68 (1.01–2.79)*	2.05 (1.02–4.09)*	1.22 (0.59–2.51)
Two or more falls	2.35 (1.31–4.22)**	3.84 (2.25–6.53)***	1.63 (0.97–2.75)
Past-month mobility device use (vs nonuse)	1.34 (0.90–1.98)	4.05 (2.54–6.44)***	3.03 (1.70–5.38)***
Having vision impairment (vs not having)	2.06 (1.27–3.34) **	1.49 (0.75–2.96)	0.72 (0.38–1.36)
Having hearing impairment (vs ot having)	1.45 (0.92–2.27)	0.79 (0.44–1.39)	0.54 (0.28–1.05)

*Notes*: CI = confidence interval; RRR = relative risk ratio. No. of observations = 1 078; population size = 4 297 568; Design *df* = 56.

*p < .05. **p < .01. ***p < .001.

Among covariates, being non-Hispanic Black, compared with non-Hispanic White was associated with a lower relative risk of experiencing both nonactivity-limiting fall worry (RRR = 0.45, 95% CI = 0.25–0.83, *p* = .011) and activity-limiting fall worry (RRR = 0.43, 95% CI = 0.25–0.73, *p* = .002); having experienced fall incidents in the past year was associated with a higher relative risk of experiencing nonactivity-limiting fall worry (single fall: RRR = 1.68, 95% CI = 1.01–2.79, *p* = .045; 2+ falls: RRR = 2.35, 95% CI = 1.31–4.22, *p* = .005) and activity-limiting fall worry (single fall: RRR = 2.05, 95% CI = 1.02–4.09, *p* = .043; 2+ falls: RRR = 3.84, 95% CI = 2.25–6.53, *p* < .001).

Education was associated with 2 types of fall worry differently: having 12+ years of formal education, compared to having <12 years of formal education, was associated with a lower relative risk of experiencing nonactivity-limiting fall worry (12 years: RRR = 0 .43, 95% CI = 0.24–0.75, *p* = .004; >12 years: RRR = 0.54, 95% CI = 0.32–0.90, *p* = .019). However, having 12+ years of formal education was associated with a higher risk of experiencing activity-limiting fall worry (12 years: RRR = 2.31, 95% CI = 1.16–4.60, *p* = .018; >12 years: RRR = 3.09, 95% CI = 1.34–7.17, *p* = .009).

Being female (RRR = 1.70, 95% CI = 1.09–2.65, *p* = .019) and having a visual impairment (RRR = 2.06, 95% CI = 1.27–3.34, *p* = .004) were only associated with a higher relative risk of experiencing nonactivity-limiting fall worry. Age 75–85 (RRR = 1.87, 95% CI = 1.03–3.39, *p* = .040; RRR = 2.00, 95% CI = 1.06–3.77, *p* = .034), compared to age 65–75, and past-month mobility device use (RRR = 4.05, 95% CI = 2.54–6.44, *p* < .001; RRR = 3.03, 95% CI = 1.70–5.38, *p* < .001) were only associated with a higher relative risk of activity-limiting fall worry.

### Associations of Fall Worry With the Pain Severity and Pain Location

Table 4 shows that severe pain was significantly associated with a higher risk of activity-limiting fall worry but not nonactivity-limiting fall worry (RRR = 2.05, 95% CI = 1.12–3.75, *p* = .020). This finding supports H2. Pain in the lower body (knee: RRR = 2.18, 95% CI = 1.29–3.70, *p* = .005; foot: RRR = 1.87, 95% CI = 1.05–3.30, *p* = .033; and leg: RRR = 1.95, 95% CI = 1.18–3.22, *p* = .010) and upper body (hand: RRR = 2.29, 95% CI = 1.38–3.81, *p* = .002; wrist: RRR = 2.51, 95% CI = 1.15–5.46, *p* = .021; shoulder: RRR = 1.77, 95% CI = 1.02–3.04, *p* = .041; neck: RRR = 2.01, 95% CI = 1.10–3.66, *p* = .024; and stomach: RRR = 2.03, 95% CI = 1.04–3.96, *p* = .038) was significantly associated with a higher risk of activity-limiting fall worry relative to having no fall worry. Pain sites in the knee (RRR = 1.78, 95% CI = 1.05–3.03, *p* = .034), hand (RRR = 1.96, 95% CI = 1.26–3.05, *p* = .003), and leg (RRR = 2.09, 95% CI = 1.12–3.91, *p* = .022) are significantly associated with a higher risk of activity-limiting fall worry relative to nonactivity-limiting fall worry. These findings partially support H3.

**Table 4.** Associations of Fall Worry With Pain Severity and Locations of Pain Sites: Multinomial Logistic Regression Results

Variable	(2) Nonactivity-Limiting Fall Worry vs (1) No Fall Worry	(3) Activity-Limiting Fall Worry vs. (1) No Fall Worry	(3) Activity-Limiting Fall Worry vs. (2) Nonactivity-Limiting Fall Worry
	RRR (95% CI)	RRR (95% CI)	RRR (95% CI)
Pain severity (vs mild)			
Moderate	1.15 (0.62–2.13)	0.85 (0.33–2.18)	0.74 (0.26–2.15)
Severe	1.17 (0.60–2.27)	2.05 (1.12–3.75)*	1.75 (0.73–4.19)
Pain sites			
1. Back pain	0.81 (0.48–1.38)	1.46 (0.84–2.55)	1.81 (0.96–3.40)
2. Hip pain	0.94 (0.59–1.49)	1.47 (0.94–2.32)	1.57 (0.87–2.83)
3. Knee pain	1.22 (0.76–1.97)	2.18 (1.29–3.70)**	1.78 (1.05–3.03)*
4. Foot pain	1.21 (0.72–2.03)	1.87 (1.05–3.30)*	1.55 (0.77–3.09)
5. Hand pain	1.17 (0.73–1.86)	2.29 (1.38–3.81)**	1.96 (1.26–3.05)**
6. Wrist pain	1.44 (0.70–2.99)	2.51 (1.15–5.46)*	1.74 (0.91–3.32)
7. Shoulder pain	1.60 (1.00–2.57)	1.77 (1.02–3.04)*	1.10 (0.55–2.19)
8. Head pain	1.19 (0.71–2.00)	1.78 (0.96–3.28)	1.49 (0.69–3.19)
9. Neck pain	1.19 (0.74–1.93)	2.01 (1.10–3.66)*	1.68 (0.90–3.16)
10. Arm pain	1.27 (0.71–2.25)	1.66 (0.86–3.21)	1.31 (0.61–2.79)
11. Leg pain	0.93 (0.53–1.64)	1.95 (1.18–3.22)*	2.09 (1.12–3.91)*
12. Stomach pain	1.24 (0.65–2.35)	2.03 (1.04–3.96) *	1.64 (0.92–2.91)

*Notes*: Multinomial logistic regression model adjusting for age, gender, race/ethnicity, education, marital status, living arrangement (living alone vs living with others), number of chronic conditions (0–1, 2–3, 4–9), cognitive status, past-year fall incidents, past-month mobility device use, and vision and hearing impairment. No. of observations = 796; population size = 3 187 588; Design *df* = 56. CI = confidence interval; RRR = relative risk ratio.

*p < .05. **p < .01. ***p < .001.

### Post Hoc Analysis: Possible Versus Probable Dementia

Post hoc analysis showed no significant differences between people with possible versus probable dementia in terms of fall worry, pain severity, and most of the pain locations. However, the probable dementia group had a significantly higher average number of pain sites than the possible dementia group (2.8 vs 2.3, *p* = .042). The 2 groups differed in several other aspects (Supplementary Tables 1 and 2): The probable dementia group was more likely to have lower cognitive functions in all 3 domains (memory, orientation, and executive function), be older, have fewer years of formal education, live with others, experience more depression/anxiety symptoms and other medical conditions, experience imbalance, have more activity limitations, and experience more leg and stomach pain. These group differences motivated the examination to determine if the patterns regarding associations between different characteristics of pain and fall worry held for people with different degrees of CI.

We repeated the multinomial logistic regressions evaluating the number of pain sites, severity, and locations within each subgroup (probable and possible dementia). Findings of these post hoc analyses revealed that the number of pain sites was associated with activity-limiting fall worry for both groups (possible dementia: RRR = 1.30, 95% CI = 1.16–1.46, *p* < .001; probable dementia: RRR = 1.13, 95% CI = 1.01–1.27, *p* = .034); however, the number of pain sites was associated with nonactivity-limiting fall worry only for the possible dementia group (RRR = 1.14, 95% CI = 1.01–1.29, *p* = .039; see results in Supplementary Table 3). The associations between severe pain and activity-limiting fall worry become not significant in both groups (possible dementia: RRR = 2.17, 95% CI = 0.97–4.84, *p* = .058; probable dementia: RRR = 2.66, 95% CI = 0.97–7.28, *p* = .057; see results in Supplementary Table 4).

We found that most of the pain location variables were only associated with activity-limiting fall worry in the possible dementia group, whereas only hand (RRR = 2.28, 95% CI = 1.11–4.68, *p* = .025) and stomach pain (RRR = 2.86, 95% CI = 1.01–8.09, *p* = .047) were associated with activity-limiting fall worry for people with probable dementia. See results in Supplementary Table 4.

The associations between covariates and fall worry in the post hoc analyses varied by cognitive status of older people with CI (see results in Supplementary Table 3): (i) being in older age groups, experiencing a single fall last year, and having vision impairment were only significantly associated with a higher risk of activity-limiting fall worry for the possible dementia group; (ii) being non-Hispanic black was only significantly associated with a lower risk of both types of fall worry for the possible dementia group; (iii) being female and being unmarried/with no partner were only significantly associated with a higher risk of fall worry for the probable dementia group. Findings about education, multiple falls in the last year, and past-month mobility device use were consistent with the major study results.

## Discussion

To the best of our knowledge, this is the first study to examine the prevalence of fall worry, associated pain characteristics, and other correlates of fall worry using a large nationally representative sample of U.S. community-dwelling older people with CI. Our study demonstrated that fall worry and pain could be elicited directly from the majority of them who were communicative.

### Fall Worry Among Community-Dwelling Older People With CI

Our findings show that fall worry is prevalent among this population despite their CI. We also found that a larger proportion of those with activity-limiting fall worry had probable dementia than those with nonactivity-limiting fall worry. It may be due to neuropsychiatric symptoms of older people with CI, such as anxiety and depression, which may decrease confidence in their physical health status and make them more afraid of falling (52). CI can also result in gait and balance impairment and visuospatial changes, such as diminished contrast sensitivity, motion detection, visual fields, and visuospatial function, which may make them worry about falls (53). Previous studies examining fall worry among those with CI have had divergent results, with some finding that greater CI is associated with an absence of fall worry (23), whereas others have observed just the opposite, that is, older people with CI reported a higher prevalence of fall worry compared to those without CI (17,27). Given these divergent results, further investigation is warranted.

### Pain Characteristics and Activity-Limiting Fall Worry

We found that the number of pain sites was significantly associated with a higher risk of activity-limiting fall worry after adjusting for participants’ sociodemographic and health characteristics. This may be attributable to the impact of multisite pain on reduced lower extremity mobility, increased extremity impairment, decreased balance, increased fall risk, and increased symptoms of anxiety and depression (54). Despite the negative impacts of multisite pain and its high prevalence, it remains an under-appreciated and understudied pain characteristic (54).

Among people who reported at least 1 pain site, we found that the association between severe pain, that is, pain that results in activity avoidance, was associated with activity-limiting fall worry but not nonactivity-limiting fall worry. Previous studies have identified significant associations between pain severity and fall worry and avoidance of activities due to fall worry among older people without dementia (55,56). Our findings show that such associations persist among community-dwelling older people with CI.

Existing studies have mainly examined the associations between pain in the lower extremity, hip, and back, and fall worry for older adults (43,57–59). We found that pain in the lower body (knee, foot, and leg) and the upper body (hand, wrist, shoulder, neck, and stomach) was associated with activity-limiting fall worry for older people with CI after adjusting covariates. Surprisingly, we did not see significant associations between fall worry and pain in the back and hip, which may be because back and hip pain may severely affect their mobility and make them more sedentary (60). It is unclear why upper body pain is associated with activity-limiting fall worry. Limited studies found that shoulder pain (61) and neck pain (62) can contribute to reduced balance. It may also be because upper body pain can increase their concerns about the inability to self-protect when they fall (63), transfer appropriately, get up safely using the upper body (64), and use mobility devices correctly (65).

### Covariates of 2 Types of Fall Worry

Our study contributes to the emerging body of literature on the correlates of 2 types of fall worry among community-dwelling older people with CI. We found that being non-Hispanic Black, compared to non-Hispanic White, was associated with a lower level of both types of fall worry. The lower likelihood of fall worry among Black older people was consistent with previous findings. However, literature suggested that Black older people in the United States were at a higher risk of experiencing CI (66) and were more likely to report risk factors associated with falling, such as physical limitations (67). The lower prevalence of fall worry among older Blacks may reflect their more extensive care networks compared to non-Hispanic Whites (68) and greater collaborations within care networks (69) given the importance of informal care partners in reducing fall worry for older people with CI (70). It may also reflect resilience as a culturally valued trait that has historically helped African American communities deal with oppression and discrimination (71). Like older people without dementia, having a fall history is associated with a higher level of both types of fall worry. Our findings suggest that despite cognitive decline, the experience of fall incidents and various fall risk factors may still contribute to fall worry (15).

Our results showed that a lower education level was associated with a higher risk of having nonactivity-limiting fall worry and a lower risk of having activity-limiting fall worry. This finding conflicted with a previous study, showing that a higher education level was associated with a lower risk of fall worry among older people with CI (18). It may be because those with higher education levels might have more resources to obtain information on how to prevent falls and adopt these fall prevention strategies (72). However, people with higher education may also have more information and access to assistance for everyday activities (eg, grocery shopping, cooking, laundry) and home- and community-based services (eg, personal care, home health) when they worry about falls. Therefore, it may be easier for them to curtail everyday activities. Another possible explanation is, in the United States, public housing rental units and subsidized private rental units are more likely to be accessible for persons with moderate mobility difficulties than owner-occupied units (73).

### Covariates Exclusively Associated With Activity-Limiting Fall Worry

Our study showed that past-month mobility device use was associated with activity-limiting fall worry. Older people may experience various challenges when using mobility devices due to their declining cognitive functions, increased cognitive demands related to attentional processing and neuromotor control, and limited insight into safe gait-aid use (74). Considering that more than 40% of community-dwelling older people with CI use devices to improve their mobility, effective interventions are urgently needed to assist them in using mobility devices safely.

### Covariates Exclusively Associated With Nonactivity-Limiting Fall Worry

We found factors exclusively associated with nonactivity-limiting fall worry, including female gender and visual impairment. One previous study identified higher fall worry among community older women with early-stage dementia (18). Our study found that women were more likely to experience nonactivity-limiting fall worry. This may be because women often learn about falls from their caregiving experiences; thus, they are more likely to be aware of the risk of falls but feel confident about adopting different fall prevention strategies (75). The relational theory of gender has also suggested that perceptions of manhood and womanhood may shape people’s experiences of health conditions (76). As the perception of manhood in Western culture is often associated with being “physically strong” and “independence,” men might be less likely to admit their concerns for falls; although the perception of womanhood is associated with being emotional and open about feelings, it could be suggested that women more readily admit their fall worry. Future studies should examine how these stereotype presumptions affect fall worry for older people with CI.

Visual impairment causes a wide range of challenges to older people’s physical function and psychological health, which are all associated with increased fall worry (77). However, visual impairment may also enable older people and their care partners to develop adaptive strategies to mitigate the negative impact of fall worry on their daily activities (78), even before the onset of CI (70).

### Differential Factors Associated With Fall Worry Among Older People With CI by Cognitive Status

In the post hoc analysis, we found the number of pain sites associated with activity-limiting fall worry in both groups. However, we found that pain at most locations was only significantly associated with activity-limiting fall worry for those with possible dementia but not probable dementia. The different patterns may be observed because of the small sample size in the probable dementia group. It may be because older people with more CI are less able to recall and interpret their experiences of pain (79).

Furthermore, we also identified differential risk factors of fall worry by their cognitive status. Only education attainment, multiple falls in the last year, and past-month mobility device use are important factors of fall worry regardless of participants’ cognitive status. It is unclear why certain factors are only relevant to the possible dementia group (age, single fall last year, vision impairment) and why other factors are only relevant to the probable dementia group (gender, marital status). These differences are puzzling with limited existing literature. One likely interpretation is that people with less CI are more aware of fall risk factors, such as age, single fall event, and visual impairment. The association between being female and nonactivity-limiting fall worry was found only in the probable dementia group. This may be because older people with CI who are female have stronger affective experiences of falls (76) and emotional responses may have been retained (15) despite their worsening CI. The associations between marital status and activity-limiting fall worry for the probable dementia group highlight the importance of spouses/partners in reducing fall worry when older people with CI increasingly need support in their everyday activities (70).

## Strengths and Limitations

The study’s strengths lie in using nationally representative data that allowed for examining the prevalence of fall worry and associations between different pain characteristics and fall worry (with and without causing activity limitations) among a large sample of community-dwelling older people with CI identified using a validated algorithm (40). The study had several limitations:

(1) Fall worry in NHATS was measured by combining 2 dichotomous responses; although simple questions can be preferable for those with CI (80), future studies may consider using measures of fall efficacy that have been validated for this population (17).
(2) The number and locations of pain in the NHATS were measured using a card listing different pain locations, which could be cognitively challenging for some. Future research may consider incorporating and validating other assessment approaches, such as the body map from the McGill Pain Questionnaire (46), asking them to point to their own body (45), health care providers’ examination (81), or proxy report (82).
(3) Few studies have assessed pain and fall worry among older people with CI; thus, a cross-sectional design represents an important first step in understanding their relationship in this population. This study’s cross-sectional design did not allow for causal inference, and thus the causal relationship between pain and fall worry requires further investigation; the current study also did not examine how pain and 2 types of fall worry were associated with fall risk reduction in the following years.
(4) As we excluded participants who used the proxy report, we might have excluded older people with more advanced dementia; Therefore, our findings may not be generalizable to those who cannot verbally report their pain and/or fall worry.
(5) Our post hoc analyses suggest cognitive status might impact the association between pain and fall worry and differential factors associated with fall worry among older people with CI with varying degrees of cognitive functions. However, the sample size for the subgroup analysis was relatively small. These preliminary findings thus require further examination.

## Implications

Despite these limitations, the findings have important research and clinical implications:

First, future studies should further examine the reasons for divergent results on the associations between CI and fall worry. The findings from our cross-sectional analysis reveal the need for future studies using a longitudinal design and a larger sample size to establish whether there are causal associations between different pain characteristics and fall worry for older people with varying degrees of cognitive functions. Our findings also suggest the need to further understand the association between upper body pain and fall worry for older people with CI. Future research should examine whether pain and fall worry predict fall events using a longitudinal study design. This work will inform future intervention studies of fall prevention interventions for this high-risk population.

Our study findings emphasize the importance of integrating pain assessment as an element of fall risk assessment and management for older people with CI and vice versa. Clinicians also need to prioritize addressing multisite and severe pain, pay attention to both upper and lower body pain, and utilize strategies to mitigate the negative impacts of pain at different locations. As pain medication is associated with increased fall risk for older people with CI (31), nonpharmacological interventions are especially essential for this population, such as music therapy, reflexology, painting and singing, cognitive–behavioral therapy, and play activity (38). So far, there are limited evidence-based programs to reduce fall worry for community-dwelling older people with CI. Exercise programs designed and tailored to them and their care partners have shown promising results (83–86). As pain is common among older people with CI and can impede their participation in exercise (87), more research is needed on incorporating innovative pain management approaches in fall prevention interventions for them.

Finally, there is a need for more investigation of other important factors associated with fall worry among older people with CI, such as gender, race/ethnicity, education, marital status, visual impairment, and the use of mobility devices. Future research should be conducted to understand the relative importance of these factors for older people with different degrees of CI. These findings will help tailor interventions for older people with CI of diverse backgrounds across a range of health and functional status.

## Supplementary Material

igad100_suppl_Supplementary_Material
