# Supplementary material for: Association Between Pain and Fall Worry Among Community-Dwelling Older People With Cognitive Impairment in the United States
Source: Innov Aging. 2023 Sep 16;7(10):igad100. doi: 10.1093/geroni/igad100 (PMC10714914; doi:10.1093/geroni/igad100)
Supplement: igad100_suppl_Supplementary_Material [file igad100_suppl_supplementary_material.docx]

**Online Supplementary Material**

NHATS 2015 older people with cognitive impairment (CI) n=1744

NHATS 2015 community-dwelling older people with CI n=1472

NHATS 2015 community-dwelling older people with CI sample who do have at least one care partner n=1150

Participants residing in nursing home or residential care facilities n=272

Participants who used proxy report n=322

NHATS 2015 people ages 65 and older n=8334

Participants who were not categorized as probable or possible dementia n=6590

Supplementary Figure 1. Flow chart of sample restrictions

**Supplementary Table 1. Sociodemographic characteristics and health status by cognitive status among community-dwelling older people with cognitive impairment**

|  | | | | | Total  (n=1150) | Possible dementia (n=706) | Probable dementia (n=444) | P-value |
| --- | --- | --- | --- | --- | --- | --- | --- | --- |
|  | | | | | %/Mean (SE) | %/Mean (SE) | %/Mean (SE) |  |
| Cognitive functions (mean, SE) | | | | |  |  |  |  |
|  | | | | Memory | 4.3 (0.1) | 4.7 (0.2) | 3.3 (0.2) | **<0.001** |
|  | | | | Orientation | 4.1 (0.1) | 4.7 (0.1) | 2.9 (0.1) | **<0.001** |
|  | | | | Executive functions | 2.5 (0.1) | 2.5 (0.1) | 2.3 (0.1) | 0.053 |
| Fall worry (%) | | | | |  |  |  |  |
|  | | | No fall worry | | 65.2 | 66.0 | 63.5 | 0.103 |
|  | | | Non-activity-limiting fall worry | | 21.1 | 22.0 | 19.2 |  |
|  | | | Activity-limiting fall worry | | 13.8 | 11.9 | 17.3 |  |
| The number of pain sites (mean, SE) | | | | | 2.4 (0.1) | 2.3 (0.1) | 2.8 (0.2) | **0.042** |
| Pain site group (%) | | | | |  |  |  |  |
|  | | No pain site | | | 26.4 | 27.4 | 24.4 | 0.407 |
|  | | Single pain site | | | 19.4 | 19.8 | 18.5 |  |
|  | | Multiple pain sites (2-13) | | | 54.2 | 52.8 | 57.1 |  |
| Age group (years, %) | | | | |  |  |  |  |
|  | | 65-74 | | | 33.9 | 39.1 | 23.6 | **<0.001** |
|  | | 75-84 | | | 41.2 | 37.9 | 47.6 |  |
|  | | 85+ | | | 25.0 | 23.0 | 28.9 |  |
| Gender (%) | | | | |  |  |  |  |
|  | | Male | | | 50.5 | 50.5 | 50.4 | 0.974 |
|  | | Female | | | 49.5 | 49.5 | 49.6 |  |
| Race/ethnicity (%) | | | | |  |  |  |  |
|  | | Non-Hispanic White | | | 65.2 | 65.7 | 64.3 | 0.275 |
|  | | Non-Hispanic Black | | | 15.1 | 16.0 | 13.3 |  |
|  | | Hispanic | | | 14.1 | 12.4 | 17.4 |  |
|  | | Other | | | 5.6 | 5.8 | 5.0 |  |
| Education (%) | | | | |  |  |  |  |
|  | | <12 years | | | 37.0 | 33.5 | 44.2 | **0.024** |
|  | | 12 years | | | 31.9 | 34.2 | 27.4 |  |
|  | | >12 years | | | 31.0 | 32.3 | 28.5 |  |
| Marital status (%) | | | | |  |  |  |  |
|  | | Married/partnered | | | 45.5 | 46.4 | 43.7 | 0.455 |
|  | | Not married | | | 54.5 | 53.6 | 56.3 |  |
| Live alone (%) | | | | |  |  |  |  |
|  | | Yes | | | 31.0 | 33.5 | 26.0 | **0.029** |
|  | | No | | | 69.0 | 66.5 | 74.0 |  |
| Past-month PHQ-4 score (mean, SE) | | | | | 2.4 (0.1) | 2.3 (0.1) | 2.8 (0.2) | **0.028** |
| No. of medical conditions (%) | | | | |  |  |  |  |
|  | | 0-1 | | | 24.1 | 27.0 | 18.5 | **0.011** |
|  | | 2-3 | | | 45.7 | 45.6 | 45.8 |  |
|  | | 4-9 | | | 30.3 | 27.4 | 35.7 |  |
| History of falls in 2015 (%) | | | | |  |  |  |  |
|  | | No fall | | | 62.5 | 63.0 | 60.8 | 0.310 |
|  | | Single fall | | | 19.9 | 20.6 | 18.4 |  |
|  | | Two or more falls | | | 17.7 | 16.1 | 20.8 |  |
| Past-month mobility device use (%) | | | | |  |  |  |  |
|  | | Yes | | | 40.4 | 38.2 | 44.7 | 0.077 |
|  | | No | | | 59.6 | 61.8 | 55.3 |  |
| Self-report balance impairment (%) | | | | |  |  |  |  |
|  | Yes | | | | 42.6 | 37.6 | 52.2 | **<0.001** |
|  | No | | | | 57.4 | 62.4 | 47.8 |  |
| Vision impairment (%) | | | | |  |  |  |  |
|  | Yes | | | | 11.1 | 9.7 | 13.9 | 0.678 |
|  | No | | | | 88.9 | 90.3 | 86.1 |  |
| Hearing impairment (%) | | | | |  |  |  |  |
|  | | Yes | | | 16.2 | 16.4 | 15.9 | 0.866 |
|  | | No | | | 83.8 | 83.6 | 84.1 |  |
| Activity limitations (Mean, SE) | | | | | 1.9 (0.1) | 1.7 (0.1) | 2.3 (0.2) | **<0.001** |

Note: SE=standard error; P values were calculated with Pearson χ^2^ tests for categorical variables and ANOVA for the continuous variables.

**Supplementary Table 2. Pain severity and pain locations by cognitive status among community-dwelling older people with cognitive impairment and at least one pain site**

|  | | All  N=835  (100%) | Possible dementia  (n=513) | Probable dementia  (n=322) | P-value |
| --- | --- | --- | --- | --- | --- |
| **Pain severity (%)** | |  |  |  |  |
|  | Mild | 24.3 | 24.3 | 24.4 | 0.342 |
|  | Moderate | 31.9 | 33.9 | 28.2 |  |
|  | Severe | 43.8 | 41.8 | 47.4 |  |
| **Pain locations (%)** | |  |  |  |  |
|  | 1. Back pain | 50.8 | 48.5 | 55.2 | 0.121 |
|  | 2. Hip pain | 25.8 | 25.7 | 26.1 | 0.896 |
|  | 3. Knee pain | 39.9 | 39.0 | 41.6 | 0.590 |
|  | 4. Foot pain | 29.5 | 29.0 | 30.2 | 0.777 |
|  | 5. Hand pain | 26.2 | 25.1 | 28.2 | 0.472 |
|  | 6. Wrist pain | 12.1 | 12.5 | 11.5 | 0.780 |
|  | 7. Shoulder pain | 33.7 | 31.8 | 37.3 | 0.152 |
|  | 8. Head pain | 19.9 | 19.8 | 20.2 | 0.905 |
|  | 9. Neck pain | 23.2 | 22.2 | 24.9 | 0.496 |
|  | 10. Arm pain | 17.0 | 15.3 | 20.1 | 0.146 |
|  | 11. Leg pain | 38.0 | 34.5 | 44.6 | **0.018** |
|  | 12. Stomach pain | 15.7 | 13.3 | 20.0 | **0.019** |

Note: P values were calculated with Pearson χ^2^ tests.

**Supplementary Table 3. Associations between pain sites and fall worry by cognitive, adjusted multinomial logistic regression**

|  | | Possible dementia | | | Probable dementia | | |
| --- | --- | --- | --- | --- | --- | --- | --- |
|  | | (2) Non-activity-limiting fall worry vs. (1) No fall worry | (3) Activity-limiting fall worry vs. (1) No fall worry | (3) Activity-limiting fall worry vs. (2) Non-activity-limiting fall worry | (2) Non-activity-limiting fall worry vs. (1) No fall worry | (3) Activity-limiting fall worry vs. (1) No fall worry | (3) Activity-limiting fall worry vs. (2) Non-activity-limiting fall worry |
|  |  | RRR  (95% CI) | RRR  (95% CI) | RRR  (95% CI) | RRR  (95% CI) | RRR  (95% CI) | RRR  (95% CI) |
| Number of pain sites | | 1.14 *  (1.01 - 1.29) | 1.30***  (1.16 - 1.46) | 1.14*  (1.02 - 1.27) | 0.99  (0.88 - 1.12) | 1.13*  (1.01 - 1.27) | 1.14  (0.99 - 1.31) |
| Age group: vs. 65-75 | |  |  |  |  |  |  |
|  | 75-85 | 0.97  (0.52 - 1.81) | 2.73*  (1.22 - 6.11) | 2.82*  (1.22 - 6.48) | 0.69  (0.25 - 1.88) | 1.32  (0.51 - 3.45) | 1.93  (0.78 - 4.77) |
|  | 85+ | 1.56  (0.78 - 3.12) | 2.58*  (1.14 - 5.83) | 1.65  (0.73 - 3.73) | 0.42  (0.17 - 1.04) | 0.65  (0.25 - 1.70) | 1.53  (0.54 - 4.31) |
| Female (vs. Male) | | 1.34  (0.76 - 2.34) | 1.43  (0.63 - 3.26) | 1.07  (0.45 - 2.54) | 2.79**  (1.37 - 5.72) | 1.20  (0.47 - 3.02) | 0.43  (0.14 - 1.34) |
| Race/ethnicity (vs. Non-Hispanic White) | |  |  |  |  |  |  |
|  | Non-Hispanic Black | 0.44*  (0.20 - 0.98) | 0.31*  (0.13 - 0.78) | 0.71  (0.26 - 1.92) | 0.43  (0.16 - 1.12) | 0.60  (0.24 - 1.47) | 1.41  (0.42 - 4.64) |
|  | Hispanic and other | 0.48  (0.21 - 1.10) | 0.98  (0.45 - 2.13) | 2.01  (0.67 - 6.07) | 1.13  (0.42 - 3.06) | 1.97  (0.69 - 5.63) | 1.73  (0.45 - 6.66) |
| Education (vs. <12 years) | |  |  |  |  |  |  |
|  | 12 years | 0.40**  (0.20 - 0.78) | 0.40*  (0.18 - 0.90) | 1.01  (0.41 - 2.48) | 0.29**  (0.13 - 0.63) | 2.25  (0.78 - 6.43) | 7.80**  (2.18 - 27.94) |
|  | >12 years | 0.54  (0.29 - 1.00) | 1.28  (0.54 - 3.02) | 2.38  (0.97 - 5.84) | 0.52  (0.21 - 1.29) | 2.37  (0.91 - 6.18) | 4.56*  (1.14 - 18.28) |
| Married/partnered (vs. Not married) | | 1.04  (0.53 - 2.06) | 0.99  (0.40 - 2.46) | 0.95  (0.37 - 2.49) | 0.91  (0.38 - 2.20) | 0.34*  (0.13 - 0.90) | 0.37  (0.12 - 1.14) |
| Live alone (vs. Living with others) | | 1.53  (0.75 - 3.09) | 1.53  (0.61 - 3.86) | 1.00  (0.47 - 2.12) | 1.22  (0.49 - 3.07) | 0.61  (0.23 - 1.62) | 0.50  (0.20 - 1.23) |
| No. of medical conditions (vs.0-1) | |  |  |  |  |  |  |
|  | 2-3 | 1.08  (0.61 - 1.94) | 0.93  (0.38 - 2.27) | 0.86  (0.31 - 2.38) | 0.99  (0.34 - 2.92) | 0.83  (0.30 - 2.29) | 0.84  (0.25 - 2.76) |
|  | 4-9 | 1.48  (0.74 - 2.98) | 1.04  (0.41 - 2.67) | 0.70  (0.23 - 2.17) | 2.00  (0.72 - 5.54) | 1.03  (0.38 - 2.78) | 0.52  (0.18 - 1.49) |
| Past-year fall incident (vs. 0 fall) | |  |  |  |  |  |  |
|  | Single fall | 1.94*  (1.07 - 3.50) | 4.08**  (1.69 - 9.88) | 2.11  (0.97 - 4.57) | 1.33  (0.53 - 3.32) | 0.45  (0.14 - 1.43) | 0.34  (0.09 - 1.21) |
|  | Two or more falls | 3.02**  (1.48 - 6.20) | 4.55***  (2.18 - 9.51) | 1.51  (0.72 - 3.14) | 1.42  (0.61 - 3.32) | 3.89***  (1.81 - 8.36) | 2.75*  (1.18 - 6.38) |
| Past-month mobility device use (vs. non-use) | | 1.44  (0.90 - 2.31) | 5.33***  (2.52 - 11.31) | 3.70**  (1.64 - 8.36) | 1.30  (0.70 - 2.40) | 2.93***  (1.62 - 5.32) | 2.26*  (1.03 - 4.97) |
| Having vision impairment (vs. Not having) | | 3.15**  (1.59 - 6.23) | 4.75**  (1.86 - 12.11) | 1.51  (0.69 - 3.29) | 1.14  (0.50 - 2.60) | 0.34  (0.11 - 1.01) | 0.30  (0.09 - 1.01) |
| Having hearing impairment (vs. Not having) | | 1.49  (0.80 - 2.79) | 0.74  (0.37 - 1.49) | 0.50  (0.24 - 1.01) | 1.32  (0.50 - 3.47) | 0.79  (0.28 - 2.21) | 0.60  (0.21 - 1.69) |
|  | | No. of observations = 668; Population size = 2,857,374; Design df = 56 | | | No. of observations = 410; Population size = 1,440,194; Design df = 55 | | |

Note:

*p < .05. **p < .01. ***p < .001.

**Supplementary Table 4. Associations between pain intensity and pain locations and fall worry by cognitive status among community-dwelling older people with cognitive impairment and at least one pain site, adjusted multinomial logistic regression**

|  | | Possible dementia | | | Probable dementia | | |
| --- | --- | --- | --- | --- | --- | --- | --- |
|  | | (2) Non-activity-limiting fall worry vs. (1) No fall worry | (3) Activity-limiting fall worry vs. (1) No fall worry | (3) Activity-limiting fall worry vs. (2) Non-activity-limiting fall worry | (2) Non-activity-limiting fall worry vs. (1) No fall worry | (3) Activity-limiting fall worry vs. (1) No fall worry | (3) Activity-limiting fall worry vs. (2) Non-activity-limiting fall worry |
|  | | RRR  (95% CI) | RRR  (95% CI) | RRR  (95% CI) | RRR  (95% CI) | RRR  (95% CI) | RRR  (95% CI) |
| **Pain severity (vs. mild)** | |  |  |  |  |  |  |
|  | Moderate | 0.97  (0.44 - 2.15) | 0.89  (0.27 - 2.92) | 0.92  (0.25 - 3.31) | 1.56  (0.55 - 4.40) | 0.98  (0.24 - 3.96) | 0.63  (0.14 - 2.75) |
|  | Severe | 1.04  (0.44 - 2.49) | 2.17  (0.97 - 4.84) | 2.08  (0.69 - 6.24) | 1.64  (0.55 - 4.89) | 2.66  (0.97 - 7.28) | 1.62  (0.46 - 5.74) |
| **Pain sites** | |  |  |  |  |  |  |
|  | 1. Back pain | 0.92  (0.52 - 1.64) | 2.18*  (1.09 - 4.36) | 2.37*  (1.11 - 5.06) | 0.49  (0.23 – 1.04) | 0.95  (0.38 – 2.38) | 1.93  (0.75 - 4.96) |
|  | 2. Hip pain | 1.22  (0.67 - 2.24) | 1.77  (0.90 - 3.50) | 1.45  (0.67 - 3.12) | 0.77  (0.37 - 1.62) | 1.30  (0.68 - 2.45) | 1.68  (0.70 - 4.05) |
|  | 3. Knee pain | 1.44  (0.84 - 2.46) | 2.29*  (1.08 - 4.87) | 1.60  (0.74 - 3.44) | 0.86  (0.30 - 2.46) | 1.84  (0.91 - 3.75) | 2.14  (0.81 - 5.67) |
|  | 4. Foot pain | 1.00  (0.52 - 1.89) | 1.93  (0.88 - 4.23) | 1.94  (0.82 - 4.55) | 1.40  (0.54 - 3.64) | 1.63  (0.68 - 3.88) | 1.17  (0.43 - 3.16) |
|  | 5. Hand pain | 1.25  (0.67 - 2.36) | 2.13*  (1.06 - 4.30) | 1.70  (0.83 - 3.46) | 1.37  (0.56 - 3.38) | 2.28*  (1.11 - 4.68) | 1.66  (0.71 - 3.87) |
|  | 6. Wrist pain | 2.02  (0.77 - 5.25) | 2.10  (0.78 - 5.64) | 1.04  (0.45 - 2.43) | 0.73  (0.13 - 4.10) | 2.39  (0.80 - 7.11) | 3.27  (0.75 - 14.31) |
|  | 7. Shoulder pain | 2.30*  (1.17 - 4.51) | 3.43***  (1.69 - 6.97) | 1.50  (0.63 - 3.53) | 1.02  (0.50 - 2.11) | 0.81  (0.39 - 1.66) | 0.79  (0.32 - 1.92) |
|  | 8. Head pain | 1.63  (0.83 - 3.20) | 2.61*  (1.06 - 6.37) | 1.60  (0.61 - 4.19) | 1.05  (0.45 - 2.46) | 0.88  (0.35 - 2.23) | 0.84  (0.28 - 2.49) |
|  | 9. Neck pain | 1.24  (0.67 - 2.28) | 2.98**  (1.35 - 6.58) | 2.41*  (1.19 - 4.87) | 1.07  (0.42 - 2.72) | 1.23  (0.51 - 2.94) | 1.15  (0.40 - 3.35) |
|  | 10. Arm pain | 1.54  (0.61 - 3.90) | 1.66  (0.55 - 5.00) | 1.08  (0.39 - 2.94) | 1.00  (0.39 - 2.56) | 1.11  (0.58 - 2.15) | 1.11  (0.38 - 3.22) |
|  | 11. Leg pain | 1.04  (0.48 - 2.26) | 2.33*  (1.10 - 4.91) | 2.24*  (1.05 - 4.78) | 0.83  (0.35 - 1.96) | 1.52  (0.84 - 2.74) | 1.83  (0.69 - 4.87) |
|  | 12. Stomach pain | 1.23  (0.55 - 2.75) | 1.83  (0.74 - 4.54) | 1.49  (0.74 - 3.03) | 1.34  (0.47 - 3.83) | 2.86*  (1.01 - 8.09) | 2.14  (0.63 - 7.26) |
|  |  | No. of observations = 513; Population size = 2,078,992; Design df = 54 | | | No. of observations = 322; Population size = 1,108,596; Design df = 49 | | |

Note:

*p < .05. **p < .01. ***p < .001. RRR= relative risk ratio; CI=confidence interval.

Multinomial logistic regression model adjusting for age, gender, race/ethnicity (non-Hispanic White, non-Hispanic Black, Hispanic and other), education, marital status, living arrangement (living alone vs. living with others), number of chronic conditions (0-1, 2-3, 4-9), past-year fall incidents, past-month mobility device use, and vision and hearing impairment**.**
